# Environmental and molecular regulation of flowering in cultivated strawberry (*Fragaria x ananassa*)

**DOI:** 10.1093/hr/uhae309

**Published:** 2024-11-06

**Authors:** Ethan Darby, Tabibul Islam

**Affiliations:** Plant Sciences Department, University of Tennessee, 2431 Joe Johnson Drive, 301 Ag & Natural Resources Bldg, Knoxville, TN 37996, USA; Plant Sciences Department, University of Tennessee, 2431 Joe Johnson Drive, 301 Ag & Natural Resources Bldg, Knoxville, TN 37996, USA

## Abstract

Cultivated strawberry (*Fragaria x ananassa*) is a globally important fruit crop that shows promise as a candidate for various methods of controlled environment production. However, a better understanding of the mechanisms of the regulation of flowering is needed, as more frequent or consistent flowering would be advantageous in controlled production. It is well understood that flowering in *F. x ananassa* responds to both photoperiod and temperature; however, the mechanism behind this response is not fully understood, particularly in perpetually flowering cultivars. While some genes of interest have been identified, a more complete model has not been established. This is largely due to the complexity of the octoploid genome and a lack of current knowledge on the mechanism of temperature response in both seasonal- and perpetual-flowering *F. x ananassa*. A starting point for developing a better model of flowering response in cultivated strawberries lies in the simpler *Fragaria vesca*, which indicates an FvCO-FvFT1-FvSOC1-FvTFL1 module for control of seasonal flowering and a lack of functional FvTFL1 responsible for perpetual flowering. However, there are some key differences when discussing *F. x ananassa’s* perpetual flowering characteristics. Recent studies have helped to elucidate some of these differences, allowing for a putative model of seasonal flowering in *F. x ananassa,* as well as indicating where further questions need to be asked regarding perpetually flowering cultivars.

## Introduction

The cultivated strawberry, *Fragaria x ananassa,* is a globally significant Rosaceous crop with an octoploid genome resulting from an accidental cross of *F. virginiana* and *F. chiloensis* [1]. Strawberries are cultivated worldwide, with China, the USA, Turkey, Mexico, and Spain consistently ranking among the highest producing countries [2]. These five countries collectively produced over 6 million tons of strawberry fruit in 2021, utilizing around 186 000 hectares of land [3]. In the USA, strawberry production is the second largest noncitrus fruit industry, valued at over 3 billion dollars annually [4].

In addition to its economic importance, one must also consider the potential health benefits strawberries may provide as a component of the modern diet. Due to the prevalence of urban sprawl, a significant portion of the US population lives in ‘food-deserts’, regions defined by low access to affordable, nutritious food [5]. Additional individuals live in areas designated as food swamps, where access to junk food is more prevalent than nutritious alternatives [6]. Either situation can result in an unbalanced diet that can contribute to obesity, diabetes, and heart disease, some of the leading causes of death in the USA [7]. While not a staple crop, strawberries can supplement mere caloric intake by providing significant levels of vitamin C and folate (58.8 g and 24 μg per 100 g serving) [8] as well as other phytonutrients that have been correlated with a decrease in rates of cardiovascular disease and cancer [9].

Despite the clear economic and nutritional impact of *F. x ananassa*, current strawberry production is isolated to small regions within producing countries, which are compatible with its unique environmental requirements. For example, in the USA, nearly 90% of commercial strawberry production occurs in California (and within California, it is largely localized to coastal counties), with the remaining 10% produced almost entirely in the state of Florida [4]. This is because the reproductive behavior of *F. x ananassa* is very sensitive to photoperiodic and temperature signals [10], making production difficult in many climates as the ambient conditions will not consistently induce flower and fruit development [11]. Unfortunately, the complex octoploid genome of *F. x ananassa* has made genetic alteration through traditional breeding difficult, making catering to its environmental needs a simpler approach to consistent production. However, the resulting localization of production makes the industry susceptible to breakdown, with current challenges including climate change, shifting legislation regarding fungicides, and increases in labor cost [12] and supply chain failure as seen during the covid-19 pandemic [13].

A sustainable solution to the climate-induced localization of strawberry production is controlled environment agriculture (CEA), which can have significant impacts on food and nutritional security. CEA can range from simple glass house technology to complex approaches such as fully automated indoor vertical farms utilizing recirculating hydroponic production, artificial LED lighting, heating and cooling HVAC systems, and robotic harvesting. CEA allows for production in areas traditionally inhospitable to *F. x ananassa,* increasing access to locally produced fresh fruit and thus decreasing food insecurity [14]. These systems can also reduce the length and complexity of current supply chains if they are properly implemented [15]. CEA technologies and practices allow for the emulation of the ideal strawberry production environment (cool nights, temperate days, and optimized day length and light quality) in places like the southwestern USA (traditionally too hot for flower induction) or the northeastern USA (by extending the possible growing season). They can also help localize food production in regions that historically have not had access to fresh produce, such as inner cities or small communities in rural areas [16].

While CEA holds promise as a tool it also comes with its own set of challenges and limitations, some of which are well covered in a recent review by Cowan et al. [17]. More particularly, the production of *F. x ananassa* in a controlled environment setting presents unique challenges due to its complex environmental needs. The primary issue is one of options, as CEA allows for the dynamic control of light intensity and quality, photoperiod duration, temperature, relative humidity, airflow, and nutrient delivery. As strawberries are sensitive to these environmental conditions, a better understanding of how each setpoint impacts a range of physiological responses, including floral initiation, fruit development, total yield, crop quality, vegetative reproduction, and disease resistance is needed before the true potential of CEA production can be reached. As CEA can have a higher cost of production than traditional field agriculture, the yield and quality of the product must be optimized to increase revenue and offset initial capital expenditure as well as the daily operational expenses [18]. Control over the transition from vegetative to reproductive tissue differentiation in the axillary meristems and apical meristems will be a key step in the optimization of CEA production of *F. x ananassa.* It will help unlock increases in fruit yield and provide control over the shift between fruit and runner (a horticultural term for stolon) production. As runners are a prominent method of clonal propagation in the strawberry industry, either type of growth can be desirable in a commercial production setting, dependent on the grower’s goals.

## Environmental control of flowering

Flowering in *F. x ananassa* is environmentally regulated by the changing of the seasons, although the same environmental conditions can result in varying outcomes depending on genetic differences. These differences, or flowering habits, are broadly classified as seasonal flowering (short day or June-bearing) or perpetual flowering (long day, everbearing, or day-neutral) [19–22]. Seasonal flowering (SF) strawberry floral induction takes place at the shoot apical meristem (SAM) in response to short days and low temperatures. Contrasting this, the prominent vegetative growths occur in response to long days and warm temperatures. During this time, the axillary buds differentiate into runners rather than branch crowns [21, 23]. Perpetually flowering (PF) cultivars have previously been referred to as everbearing, long-day, and day-neutral plants. This is because they will continue floral initiation (everbearing) while desirable conditions are met (long days), resulting in continuous fruit production across the warmer months. The term day-neutral adds confusion as it was traditionally grouped as a third habit, but according to Sønsteby and Heide [24], these plants are better classified as long day, because sufficiently short days can repress flowering in these cultivars.

Strawberry plant architecture follows a rosette pattern, with a central SAM and leaves that emerge in a spiral fashion along the central axis [25]. Axillary meristems form at each internode along this central axis. These axillary meristems in turn can differentiate into stolons or branch crowns, the latter of which contain their own apical meristems that terminate their development as an inflorescence. Due to this unique architecture, an important aspect of the response of *F. x ananassa* to environmental stimuli is the tradeoff seen between flower production and runner production. During development, each axillary meristem can become a new branch crown (resulting eventually in a terminal inflorescence) or a new runner (used heavily in horticultural clonal propagation) [25]. In this way, the ultimate horticultural productivity of the plant can be increased by encouraging branch crown development, as each branch crown can increase the total number of inflorescences present on the plant. This also means that when considering environmental stimuli, one must determine if the effect of the stimuli in decreasing or increasing floral development is occurring before or after the differentiation of axillary meristematic tissue. In other words, care must be taken to discriminate between conditions that affect floral initiation, or the differentiation of axillary meristems, and those that merely repress later development of previously differentiated tissue. Conditions that impact development at the point of axillary meristematic differentiation typically produce a tradeoff, resulting in either more runners or more inflorescences. As this review focuses on floral initiation, and not the later processes of fertilization and fruit development, it will primarily focus on the environmental stimuli that impact meristematic differentiation.

### Photoperiod and temperature responses of seasonal flowering *F. X ananassa*

While variation in response to photoperiod exists between cultivars, the general trend in SF *F. x ananassa* is that shorter photoperiods induce floral initiation. The SF cultivar ‘Korona’ has been evaluated extensively, and both the photoperiod duration and number of days at a given photoperiod influence its floral initiation. Photoperiods that are 15 hours or longer prevent flowering in this cultivar, with a study showing no flower development in plants exposed to 16-, 20-, or 24-hour photoperiods [26]. Another study showed failure of floral induction in plants exposed to 15-hour photoperiods while plants exposed to 13.5-hour photoperiods produced induction equivalent to that produced by a 12-hour photoperiod [27].

Both studies also evaluated the effect of the duration of short-day exposure, finding that additional days at the short photoperiod increased the number of flowers produced, and research by Verhuel et al. [26] shows that at least 21 days of short photoperiods are required to initiate flowering in ‘Korona’. This confirms research performed by Sønsteby and Nes [28] that determined that increasing the number of short days (8-hour photoperiod) from 16 to 24 increased total flower production in ‘Korona’, although an additional 8 days was shown to decrease flowering under some conditions. While not true for every SF cultivar, similar increases in floral production in response to increases in the duration of short-day exposure have been seen in ‘Elsanta’ [28], as well as ‘Chandler’, ‘Jewel’, ‘Earliglow’, and ‘Seneca’ [29].

Temperature is another major factor in the environmental regulation of strawberry flowering. In SF cultivars of *F. x ananassa*, lower temperatures can help to induce floral initiation. For example, temperatures of 15°C initiate more flowers under short-day conditions in the cultivars ‘Korona’ and ‘Elsanta’ when compared to 21°C [28]. The same study showed that temperatures of 9 and 15°C can induce floral initiation in ‘Bounty’ without exposure to short days, while floral initiation does not start at 21°C until 16 weeks of exposure to short days have occurred. This finding is further confirmed in ‘Korona’ plants grown under temperatures ranging from 12 to 30°C daytime temperatures, in which a linear decrease in total flowers per plants was seen as temperature increased [26]. Temperature responses can be further complicated by the differential between day and night temperature, and for ‘Korona’ and ‘Elsanta’, a day/night treatment of 18/12°C provided optimal flowering, while night temperatures of 6°C or day temperatures of 12 or 27°C reduced flowering [30].

As briefly mentioned before, some interactive effects are seen between temperature and photoperiod, and the resulting phenotypic effect again varies from cultivar to cultivar. In the case of ‘Korona’, ‘Elsanta’, and ‘Bounty’, this interaction seems to function as a sort of sliding gradient, where a given temperature alters the number of short days required to induce flowering [28]. More specifically, warmer temperatures seem to increase the number of short days required while cold temperatures reduce this requirement. Some of this interplay has been explored in *F. vesca*, a wild diploid relative of *F. x ananassa,* where exposure to temperatures of 9°C can induce floral induction regardless of photoperiod, while temperatures above 21°C prevented floral initiation across all photoperiods [31]*.* This indicates that the ‘sliding gradient’ can be pushed all the way to either extreme. This same study showed that under moderate temperatures (between 15 and 18°C), short photoperiods play a role in floral initiation, with 18°C temperatures requiring shorter day lengths (14 hours) than plants grown at 15°C (16 hours).

### Photoperiod and temperature responses of PF *F. X ananassa*

PF cultivars are also responsive to photoperiodic and temperature signals, but their common trend is that longer days continuously induce floral initiation. This is demonstrated in the cultivars ‘Flamenco’, ‘Ridder’, ‘Rita’, and ‘Rondo’, which all begin floral initiation rapidly after exposure to 18-hour days following a four-week period of 12-hour days [24]. As PF *F. x ananassa* plants originate primarily from two backgrounds, it is important to note that this trend also holds true for PF plants that originate from crosses of SF *F. x ananassa* and *F. virginiana ssp. glauca,* such as ‘Hecker’ [32]. The critical photoperiod for everbearing cultivars does vary by genotype, and photoperiods as short as 14 hours can reinduce flowering in ‘Summerberry’ [33], while another six everbearing cultivars show floral induction at 16 hours with little to no flowering occurring when grown at 12 hours [34].

While photoperiodic responses in PF plants are generally reversed from those seen in short-day cultivars, temperature responses remain similar. Early work by Durner et al. [35] showed that increasing day/night temperatures from 18/14 to 30/26°C decreased flower count and increased runner count in ‘Hecker’, ‘Tristar’, ‘Ourown’ and ‘Ozark Beauty’. Similarly, when ‘Flamenco’, ‘Ridder’, ‘Rita’, and ‘Rondo’ were grown under sub-optimal photoperiods flowering could still occur if temperatures remained at 15 or 21°C but stopped if temperatures rose to 27°C [24]. Another study with the cultivar ‘Everest’ showed that while high daytime temperatures (26°C) reduce yield, this effect can be offset by cool nighttime temperatures (13°C) [36]. These yield reductions are partially explained by high temperature’s impact on primary metabolic function. One study found that the optimal photosynthetic temperature for ‘Favori’ was between 15 and 21°C, although significant plasticity was seen depending on photoperiod length and irradiance level [37].

**Figure 1:**
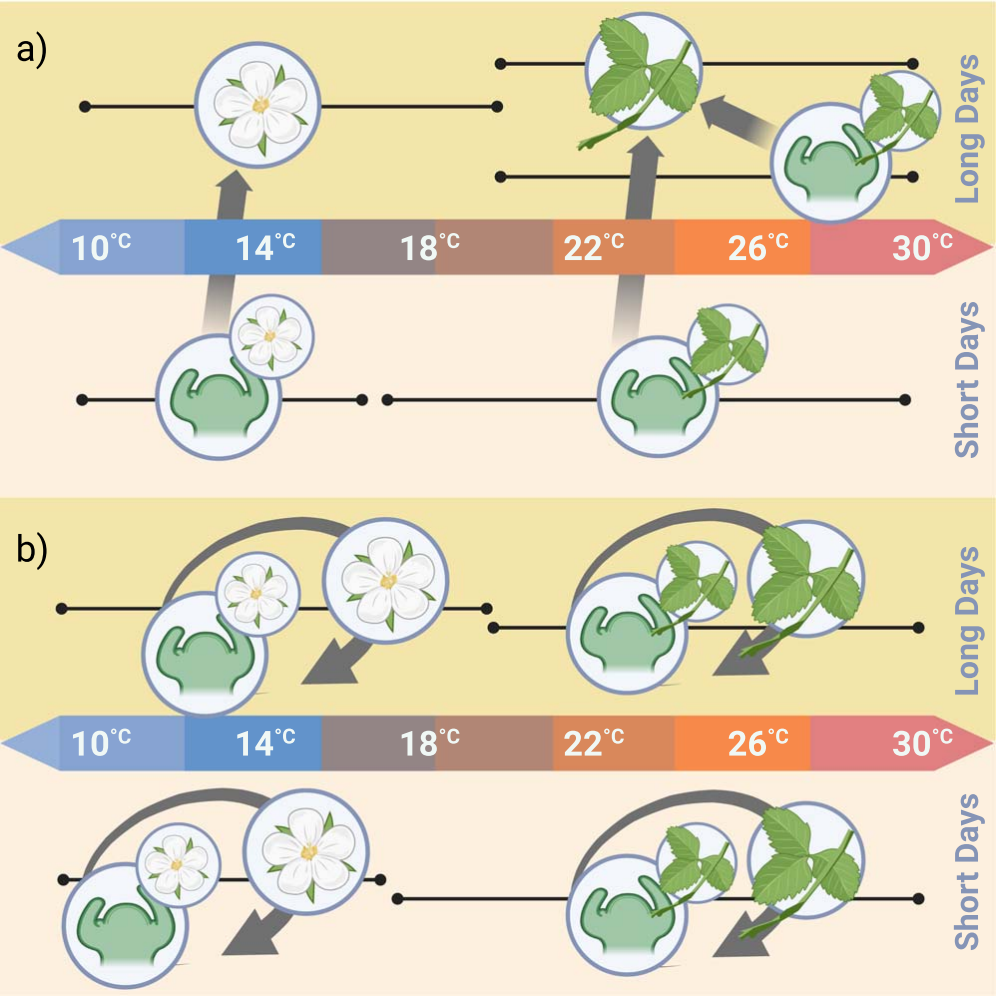
The reproductive response of both (a) seasonal- and (b) perpetual-flowering *F. x ananassa* are illustrated. Black lines with a dot represent general ranges of response to temperature at either end. Meristematic differentiation is represented by a tissue type superimposed over a meristem. Arrows represent development of a meristem into a tissue over time. In seasonal flowering cultivars, apical meristematic differentiation into inflorescence structures happens during cool, short days (a). This differentiated tissue then develops as days lengthen and temperatures rise. At warmer temperatures and longer days only, vegetative tissue is produced. In PF cultivars apical meristematic differentiation into floral tissue and its subsequent growth can occur at cooler temperatures regardless of day length. Long days are required to continue this floral differentiation and development as temperatures rise. Once temperatures are too warm only vegetative growth occurs (b).

As in the case of SF plants, and perhaps to a greater degree, PF cultivars also respond to the interaction between temperature and photoperiod, as illustrated in Fig. 1. While long photoperiods are preferable, flowering can still occur under short-day conditions if daytime temperatures remain at 21°C or lower [24]. Additionally, two PF cultivars (‘Hecker’ and ‘Summerberry’) completely stopped flowering when grown under an 8-hour photoperiod and a 30/25°C day/night temperature, but flowering reinitiated when the photoperiod was lengthened to 24 hours, or when temperatures dropped to 20/15°C [32]. These studies, as well as an additional report by Nishiyama and Kanahama [33], indicate that it is a combination of warm temperatures and short photoperiods that is particularly repressive to flowering in PF cultivars.

### Light quality as a factor in photoperiodic response

An additional cue that may control flowering in *F. x ananassa* is the quality, or spectrum, of the provided light. As light is often provided artificially in CEA agriculture and as such is narrower in bandwidth than solar irradiance, it is important to ensure that the wavelengths of light used have the intended physiological effect. Some research shows that extending the photoperiod with a 1:5 ratio of far-red:blue light significantly increased floral induction in PF strawberries (‘Albion’), including floral induction outside of the primary crown [38]. Additional research shows that SF cultivars are impacted by light quality as well, with *F. x ananassa* ‘Festival’ flowering despite a period of long, warm days due to a high ratio of far-red:red light, likely by way of phytochrome activation [39].

## Regulatory mechanisms of flowering in responses to photoperiod and temperature

### Mechanisms controlling photoperiodic responses

The physiological response of *F. x ananassa* to photoperiod and temperature signals can be seen in changes in growth rate, axillary meristematic tissue differentiation (runner or branch crown production), flower initiation, and total fruit yield. The plant regulates these changes through various signal cascades [21, 22]. While photoperiod and temperature regulations have been studied extensively in cultivated strawberry at the physiological level, the molecular mechanisms of flowering have been primarily studied in diploid strawberries. The understanding of molecular regulation in the notoriously complex octoploid strawberries is still in its infancy. The genetic analysis of the perpetual flowering characteristic in the *F. vesca* (diploid) and *F. x ananassa* (octoploid) revealed different loci in each species, indicating that differences in flowering regulatory mechanisms may exist among them [19, 20, 40, 41]. In *F. x ananassa,* the PF trait, QTL FaPFRU, has been identified; however, the underlying genes are different from the TERMINAL FLOWER 1 (TFL1) gene seen in *F. vesca*, suggesting the existence of the complex PF regulatory mechanism in octoploid strawberries. In order to better control flowering in cultivated strawberries, molecular regulations need to be characterized in both SF and PF cultivars of octoploid strawberry.

In general, plants’ first step in perceiving changes in photoperiod are photoreceptors, particularly phytochromes and cryptochromes. These proteins undergo conformational changes in response to light and slowly revert in its absence. This protein-encoded information is then utilized to measure day length in conjunction with the B-Box transcription factor CONSTANS (CO), a recognized component of the photoperiod measurement system [42, 43]. CO is expressed on a rhythmic circadian cycle [44], and its expression naturally peaks once every 24 hours. Under long-day conditions, this peak occurs while the photoreceptors are exposed to light, leading to post-transcriptional activation of CO [42]. This activation of CO triggers the induction of the flowering integrator genes FLOWERING LOCUS (FT) and SUPPRESSOR OF CONSTANS 1 (SOC1). FT and TFL1 interact with the bZIP transcription factor FD through 14–3-3 proteins, which regulate the transcriptional activation of the floral identity genes [45]. FT interactions with FD via 14–3-3 form a transcriptional activator complex for the floral identity genes and promote flowering. On the other hand, TFL1 competes with FT for FD interaction, leading to a repressor complex for flowering and promoting vegetative growth [22–26, 42–44].

The module CO-FT-SOC1-TFL1 works in both woodland and cultivated strawberry flowering regulation in response to environmental cues. However, the final outcome varies depending on the flowering habit of the specific strawberry cultivar, be it seasonal or perpetual. For instance, in SF *F. vesca*, the FvCO-FvFT1-FvSOC1 module activated under long days represses flowering by promoting the expression of the flowering suppressor FvTFL1 [22]. However, overexpression of FvCO in PF *F. vesca*, which lacks functional FvTFL1, induces the FvFT1 expression in the leaf that correlates with the upregulations of the FvSOC1 and floral meristem identity genes APETALA1 (FvAP1) at the SAMs and eventually promotes flowering [46]. Similarly, in SF *F. x ananassa*, a correlation has been observed between the upregulation of the FaCO and FaFT1 genes in leaf tissue and FaSOC1 and FaTFL1 in crowns during long-day conditions [23]. However, their role in the PF- *F. x ananassa* is poorly understood and needs further investigation.

Functional genomics approaches have recently been taken to characterize the role of FaCO and FaSOC1 in the cultivated strawberry [23]. Overexpression of the FaCO in the SF-cultivated strawberry ‘Camarosa’ has not significantly affected flowering and vegetative growth in short-day conditions. It has been revealed that overexpression of FaSOC1 repressed expression of the FaFT1-FaFT3 genes and the flowering identity-related genes FaLFYa, FaAP1, and FaFUL in the crown, which coincide with FaTFL1 gene upregulation. However, the authors suggest that FaTFL1 independent suppression of the FTs might exist in cultivated strawberries, as the expression patterns of FaTFL1 lack consistency among the different transgenic lines of overexpressed FaSOC1 [23]. Previously, the FaTFL1 function as a flower repressor was functionally characterized in SF-octoploid strawberries by knock-down, which promotes flowering and induces the PF traits [47]. Moreover, the overexpression of the FaSOC1 shows more vegetative growth and runner formation; however, it is suggested to be independent of the gibberellins (GAs) pathways as GAs biosynthesis gene pathways have not been markedly affected in both leaves and crowns [23]. The authors did not explore the accumulation patterns of various GAs, which might provide further insights into SOC1 and GA involvement in octoploid strawberry’s vegetative growth and runner formation.

**Figure 2:**
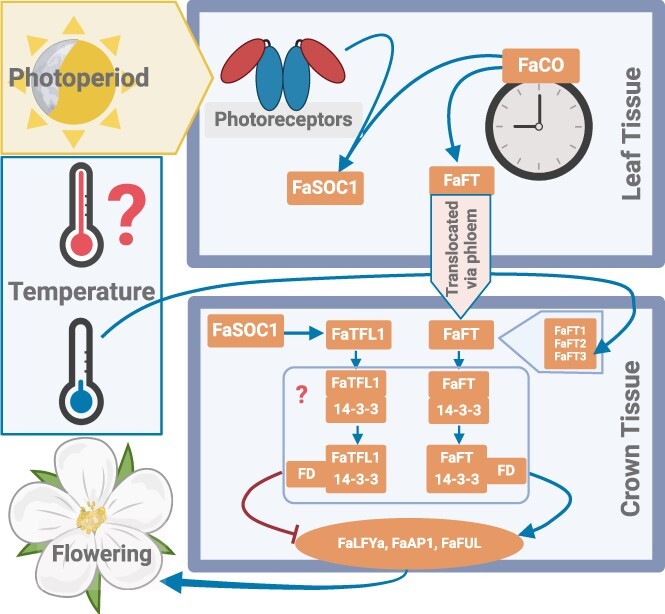
Putative floral regulatory molecular mechanism involved in the response of seasonal flowering *F. x ananassa* to photoperiod and temperature. Arrows indicate an upregulating or activating effect, while lines with blunt ends represent a repressive effect.

### Mechanisms controlling temperature responses

Compared to the body of work on the mechanisms behind photoperiodic responses, the mechanisms controlling temperature responses in *Fragaria* are less understood. Expression of *FvTFL1* is suppressed in *F. vesca* by temperatures lower than 13°C, resulting in flower initiation regardless of day length [48]. However, this regulation was not shown to proceed by way of *FvFT1* or *FvSOC1*, as is the case in the photoperiodic regulation of *FvTFL1*. One study found that *FaTFL2* was a more promising candidate for long-day/high-temperature floral repression in *F. x ananassa* ‘Tochiotome’ strawberry and that this gene was more responsive to temperature than to day length [49]. Another piece of the puzzle is further elucidated by the role of *FaFT3*, which accumulates heavily in the SAM under conditions that downregulate the expression of *FaTFL1* (short days and/or cool temperatures) and is more sensitive to temperature than photoperiod [50]. Overall, it seems that genes in the same families as those that regulate photoperiodic responses play a role in temperature response, possibly due to past duplication and subsequent divergence of function.

### Putative model of photoperiodic and temperature regulation of flowering

Figure 2 illustrates a putative model describing the effect of photoperiod and temperature on flowering in SF *F. x ananassa*. Current knowledge suggests that the FaCO-FaFT1-FaSOC1-FaTFL1 module exists in *F. x ananassa*. However, their intricate regulatory signaling cascades seem less straightforward than those observed in *F. vesca*. The perpetual flowering trait in cultivated strawberries is more complex and the regulatory elements involved in flowering and/or runnering need further characterization.

A study by Muñoz-Avila et al. [23] characterized for the first time the role of *FaCO* and *FaSOC1* in flowering and vegetative growth in cultivated strawberries. Overexpressed *FaCO* plants have not shown changes in flowering and/or runnering in short-day conditions. This indicates that additional photoperiodic regulatory elements might exist that would be responsible for the repression of flowering. Additionally, long day mediated posttranscriptional modifications of FaCO might be key for the FaCO-dependent flowering repression. On the other hand, *FaSOC1* conserved its function as a flower repressor among the diploid and octoploid strawberries, but downstream signals show differences. In fact, overexpression of *FaSOC1* did not alter the GAs biosynthetic genes, indicating GA-independent vegetative growth might exist and/or *FaSOC1* might work on the common downstream signaling genes of GAs. Future studies are needed to elucidate the molecular circuits that are responsible for the photoperiodic and temperature-mediated regulation of flowering in cultivated strawberries.

As previously stated, this putative model is a starting point for describing the seasonal flowering regulation of *F. x ananassa.* However, there is further work to be done elucidating the impact of temperature in flowering regulation. Even more elusive is a model of the behavior of PF cultivars in response to environmental cues. While this review does not aim to establish a putative model for PF cultivars, a study by Bradford et al. [51] provides a good starting point for understanding their behavior. In short, below a specific and cultivar-dependent temperature, a PF cultivar will flower regardless of photoperiod. Once this temperature is surpassed, photoperiod becomes an important controller of flowering, with long days required for continued flower production. Unfortunately, this control differs from the simple FvTFL1 deletion seen in perpetual flowering *F. vesca* accessions and further mechanistic study is required to explain the observed phenotypic responses.

A better understanding of these molecular models will allow for the creation of more informed setpoints in CEA strawberry production. Coincidentally, the precise control of environmental parameters provided by CEA tools will be key to elucidating these intricate models. Beyond the obvious impact of providing more informed environmental setpoints, the further elucidation of these molecular models will allow for the future integration of biotechnology and CEA. Molecular marker assisted selection, gene-editing, and RNAi technologies will all rely on understanding the function of each gene and its importance in the context of CEA production.

## Data Availability

Not applicable.
